# Machine Learning and Deep Learning CT-Based Models for Predicting the Primary Central Nervous System Lymphoma and Glioma Types: A Multicenter Retrospective Study

**DOI:** 10.3389/fneur.2022.905227

**Published:** 2022-08-30

**Authors:** Guang Lu, Yuxin Zhang, Wenjia Wang, Lixin Miao, Weiwei Mou

**Affiliations:** ^1^Department of Hematology, Shengli Oilfield Central Hospital, Dongying, China; ^2^Department of Neurosurgery, Guangrao County People's Hospital, Dongying, China; ^3^GE Healthcare, Shanghai, China; ^4^Department of Medical Imaging Center, Shengli Oilfield Central Hospital, Dongying, China; ^5^Department of Pediatrics, Shengli Oilfield Central Hospital, Dongying, China

**Keywords:** computed tomography, glioma, primary central nervous system lymphoma, machine learning (ML), deep neural netorks

## Abstract

**Purpose and Background:**

Distinguishing primary central nervous system lymphoma (PCNSL) and glioma on computed tomography (CT) is an important task since treatment options differ vastly from the two diseases. This study aims to explore various machine learning and deep learning methods based on radiomic features extracted from CT scans and end-to-end convolutional neural network (CNN) model to predict PCNSL and glioma types and compare the performance of different models.

**Methods:**

A total of 101 patients from five Chinese medical centers with pathologically confirmed PCNSL and glioma were analyzed retrospectively, including 50 PCNSL and 51 glioma. After manual segmentation of the region of interest (ROI) on CT scans, 293 radiomic features of each patient were extracted. The radiomic features were used as input, and then, we established six machine learning models and one deep learning model and three readers to identify the two types of tumors. We also established a 2D CNN model using raw CT scans as input. The area under the receiver operating characteristic curve (AUC) and accuracy (ACC) were used to evaluate different models.

**Results:**

The cohort was split into a training (70, 70% patients) and validation cohort (31,30% patients) according to the stratified sampling strategy. Among all models, the MLP performed best, with an accuracy of 0.886 and 0.903, sensitivity of 0.914 and 0.867, specificity of 0.857 and 0.937, and AUC of 0.957 and 0.908 in the training and validation cohorts, respectively, which was significantly higher than the three primary physician's diagnoses (ACCs ranged from 0.710 to 0.742, *p* < 0.001 for all) and comparable with the senior radiologist (ACC 0.839, *p* = 0.988). Among all the machine learning models, the AUC ranged from 0.605 to 0.821 in the validation cohort. The end-to-end CNN model achieved an AUC of 0.839 and an ACC of 0.840 in the validation cohort, which had no significant difference in accuracy compared to the MLP model (*p* = 0.472) and the senior radiologist (*p* = 0.470).

**Conclusion:**

The established PCNSL and glioma prediction model based on deep neural network methods from CT scans or radiomic features are feasible and provided high performance, which shows the potential to assist clinical decision-making.

## Introduction

Primary central nervous system lymphoma (PCNSL) and glioma are two common kinds of malignant primary tumors (1). PCNSL consists of about 2% of brain tumors, with an increasing incidence over the past decades (2). PCNSL is an aggressive type of extra nodal lymphoma without coexisting systemic disease at diagnosis (3). Glioma is the most aggressive primary malignant brain tumor in adults, accounting for about 15% of brain tumors (4). The two brain tumors can be identified, localized, and characterized using conventional techniques, such as computed tomography (CT) and magnetic resonance imaging (MRI). It is important to differentiate PCNSL from glioma since treatment options are vastly different for the two diseases. Patients with PCNSL usually have a good response to noninvasive treatments, such as chemotherapy, target therapies, and whole brain radiation treatment (5). Resection provides no therapeutic benefit and is reserved only for rare cases of neurologic deterioration due to brain herniation. But for patients with glioma, the standard treatment is the invasive maximum safe surgical resection followed by concurrent chemo-radiotherapy (6). Therefore, preoperative differentiation of PCNSL and glioma is clinically critical to guide neurosurgical treatment strategies, avoid unnecessary and potentially harmful surgery, and thus optimize patient outcomes, quality of care, and cost-effectiveness (7).

Imaging has a central role in the differentiation task of PCNSL and glioma, especially for patients whose lesion pathology cannot be obtained by puncture. However, the two diseases are challenging to differentiate based on radiology alone since they share overlapping imaging characteristics. A simple and accurate method is needed to identify PCNSL and glioma. There are multiple previous studies attempted to distinguish the two tumors. One way is to use advanced imaging techniques, such as different types of MR perfusion (8, 9), diffusion-tensor imaging (DTI) (10), and dynamic CT perfusion (11). These advanced methods have been assessed with modest success but require additional expense, time, and radiation and may not be performed routinely for every patient. Most recently, machine learning (ML) and deep learning (DL) have been applied in correctly diagnosing PCNSL and glioma. Radiomics is one successful method that extracts high-dimensional quantitative features from medical images using data-characterization algorithms and provides the information that represents the underlying pathophysiology that is difficult to acquire by visual inspection (12, 13). Suh et al. demonstrated that the diagnostic performance of MR radiomics-based machine-learning algorithm for differentiating PCNSL from atypical glioma yielded a better diagnostic performance than human radiologists and ADC values on a single medical center (14). Kunimatsu et al. developed an ML-based image classifier to differentiate between GBM and PCNSL using texture features from contrast-enhanced T1-weighted images although the prediction accuracy was only 75% on the test data (15). Yun et al. revealed that a combination of radiomic features and multilayer perceptron (MLP) network classifier served a high-performing and generalizable model for the two tumors classification tasks on a small MR dataset (16). Bathla et al. compared the predictive performance of various ML techniques to differentiate between PCNSL and glioma using a combination of various feature selection and ML algorithms on several MR sequences (17). Xia et al. (18) investigated the use of CNN model to differentiate between PCNSL and glioma without delineation from 289 MRI scans and proved that the proposed model was comparable to the radiomic models and radiologist.

Prior studies have shown the MR radiomics-based or MRI scan-based methods could successfully differentiate between PCNSL and glioma. However, some patients are not suitable for MR checks, such as critically ill patients with impaired consciousness and patients with implantable medical devices, including pacemakers. For these patients, CT check is routinely performed. CT is also a cost-effective check, and the scan time is short. To our knowledge, the effectiveness of ML or DL CT-based models for predicting the two tumors has been rarely explored. On the other hand, the amount, type, completeness, and diversity of data determine the performance of classification models. The results from multicenter trials are more representative for future clinical practice. Therefore, the aim of our study was to explore various machine learning and deep learning methods based on radiomic features extracted from CT scans or raw CT scans to predict PCNSL and glioma types and compare the performance of different models on multicenter data. For the end-to-end CNN model, we also employed visualization techniques to superimpose heatmaps which explained the important regions for making decision.

## Materials and Methods

### Study Population

**Figure 1:**
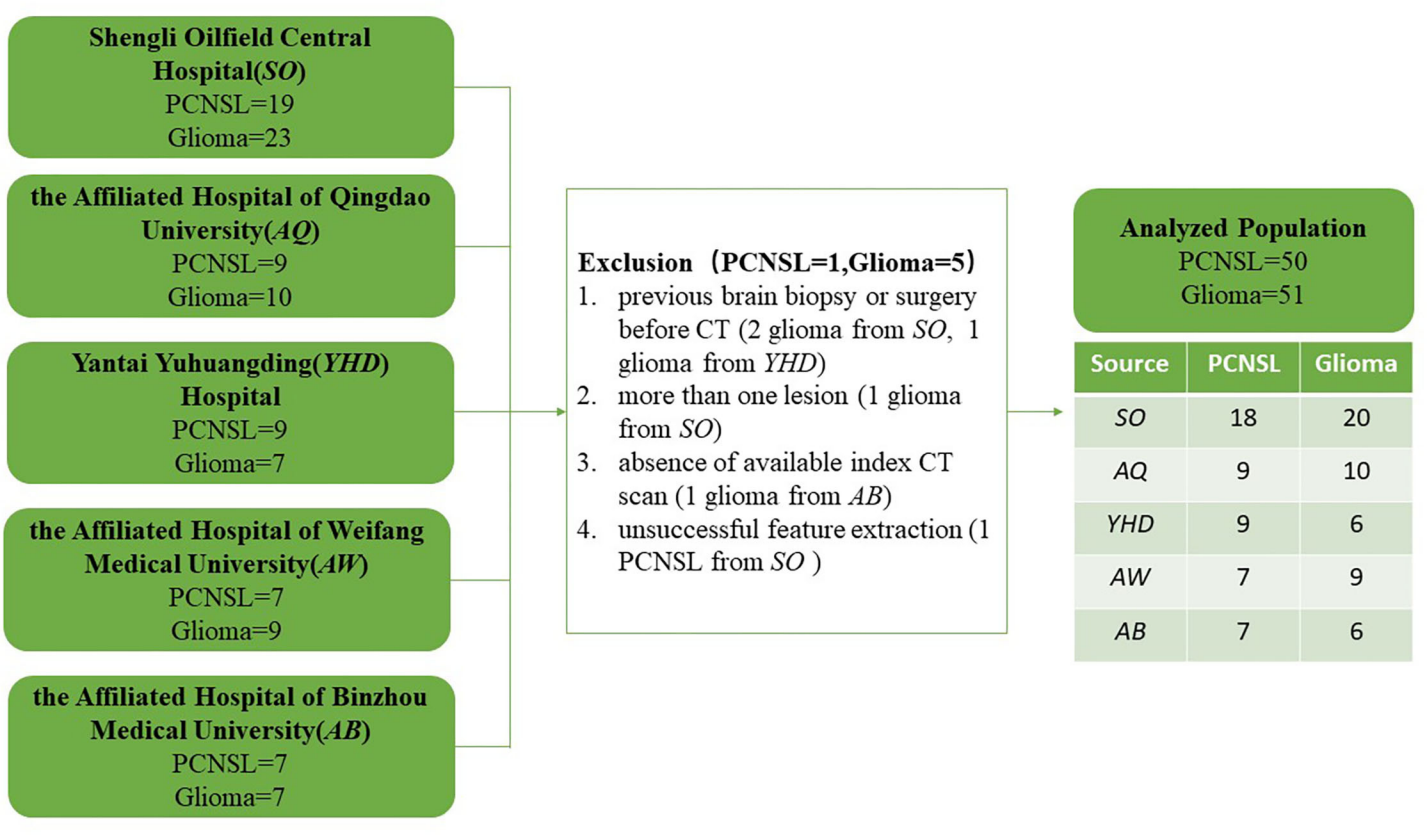
Detailed inclusion and exclusion flowchart.

The retrospective study complied with the Declaration of Helsinki (2000) and was approved by the Independent Ethics Committees of the Shengli Oilfield Central Hospital, the Affiliated Hospital of Qingdao University, Yantai Yuhuangding Hospital, the Affiliated Hospital of Weifang Medical University, and the Affiliated Hospital of Binzhou Medical University. The requirement for informed patient consent was waived. Patients who underwent brain CT and pathologically proven diagnosis of PCNSL and glioma between 1 August 2015 and 31 December 2020 were recruited from the electronic medical records. All 51 identified PCNSL cases and 56 randomly selected glioma cases were assessed for the exclusion and inclusion criteria. The exclusion criteria included the following: (1) previous brain biopsy or surgery before CT (3 glioma cases), (2) more than one lesion (1 glioma case), (3) the absence of available index CT scan (1 glioma case), and (4) unsuccessful feature extraction (1 PCNSL case). Finally, the cohort consisted of 50 PCNSL and 51 glioma who were successfully assessed (Figure 1).

### Image Acquisition

All examinations were conducted on four CT scanners: two dual-source CT scanners with ≥ 64-detector rows (Somatom Definition Flash/Force; Siemens, Forchheim, Germany) and two multi-detector rows (Optima 660, GE Healthcare, Milwaukee, WI, USA; Philips iCT 256, Philips Healthcare, Amsterdam, Nederland), following the guidelines of head CT. The tube voltage of the CT scan was 120 kV and the tube current was 200–250 mAs. Axial images were reconstructed with as slice thickness of 5 mm, spacing of 5 mm, and the matrix of 512 × 512 mm. The CT images were reconstructed with a standard kernel. The CT images were transferred to an external workstation (Syngo MMWP VE 36 A; Siemens Healthcare, Forchheim, Germany) for further postprocessing.

### Image Preprocessing

**Figure 2:**
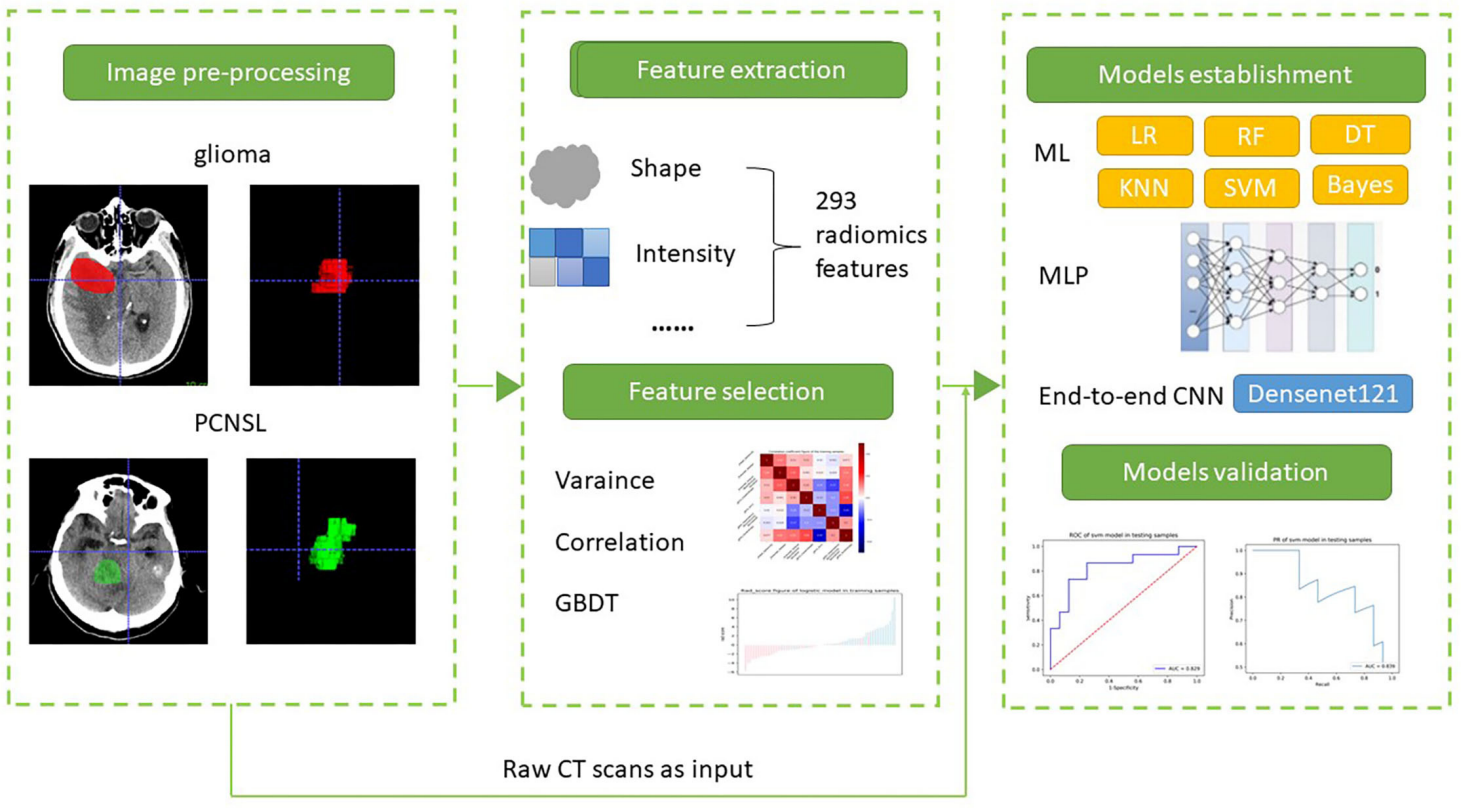
The workflow of this study. (1) Image processing, (2) feature extraction, (3) machine learning and deep learning for differentiation PCNSL from glioma.

The overview of the study workflow is provided in Figure 2. A radiologist (5 years of experience in radiology) from Shengli Oilfield Central Hospital reviewed the CT images and manually delineated the brain tumors at the axial site using MITK software version 2018.04.2 (www.mitk.org). The marked regions of interest (ROIs) were confirmed by another senior neuroradiologist (10 years of experience in radiology) who were blinded to the assessment. For the end-to-end CNN method, we converted 3D CT scans to 2D slices to overcome the small number of data points.

### Feature Extraction

Radiomic features were extracted using an open-source python package, Pyradiomics 2.2.0 (https://pyradiomics.readthedocs.io/en/latest/index.html) (19). The binWidth was set to 25 and the interpolator was “sitkBSpline.” The resampledSpacing was (1, 1, 1). In total, 293 radiomic features of each patient were extracted, which included 18 first-order histogram features, 24 gray-level co-occurrence matrix features, 14 3D-shape features, 14 gray-level dependency matrix features, 16 gray-level size zone matrix features, 16 gray-level run length matrix features, 5 neighboring gray tone difference matrix features, and 186 Laplacian of Gaussian (LoG_sigma = 2.0/3.0_) features.

### Feature Selection

We preprocessed the data and normalized the extracted features. When the data value exceeded the range of mean and standard deviation, the median of specific variance vector was used to replace the outliers. In addition, we standardized the data in a specific interval.

Due to the high dimension of the possible feature sets to be used relative to the sample size and highly correlated variables, feature selection is generally considered a critical piece of the model building process and could reduce overfitting in further differentiation model. A total of three feature selection methods were considered. The analysis of variance (ANOVA) threshold of 1.0 was applied first and 57 features remained. Then, we used filter model correlation analysis with a threshold of 0.7 and obtained 21 features. Finally, an embedded model gradient boosting decision tree (GBDT) was implemented and 7 features were selected. The feature selection process was performed using Python (version 3.5.6).

### Machine Learning Models Establishment and Validation

We randomly divided the patients into the training (*n* = 70, 35 PCNSL and 35 glioma) and validation (*n* = 31, 15 PCNSL and 16 glioma) sets by a ratio of 7:3. Then, we established six different radiomic models from the established optimal feature subsets of the training dataset using logistic regression (LR), random forest (RF), decision tree (DT), k-nearest neighboring (KNN), support vector machine (SVM), and naïve Bayes. The hyper-parameters of the RF, DT, KNN, and SVM were automatically selected by search method.

Models were trained with the training set using the 5-fold cross-validation method, and estimation performance was evaluated within the validation set. The performance of different models was assessed using the area under the receiver operating characteristic curve (AUC), accuracy (ACC), sensitivity, specificity, a positive predictive value (PPV), and a negative value (NPV). All machine learning methods were performed using Python (version 3.5.6).

### Radiomic-Based Deep Learning Models

**Figure 3:**
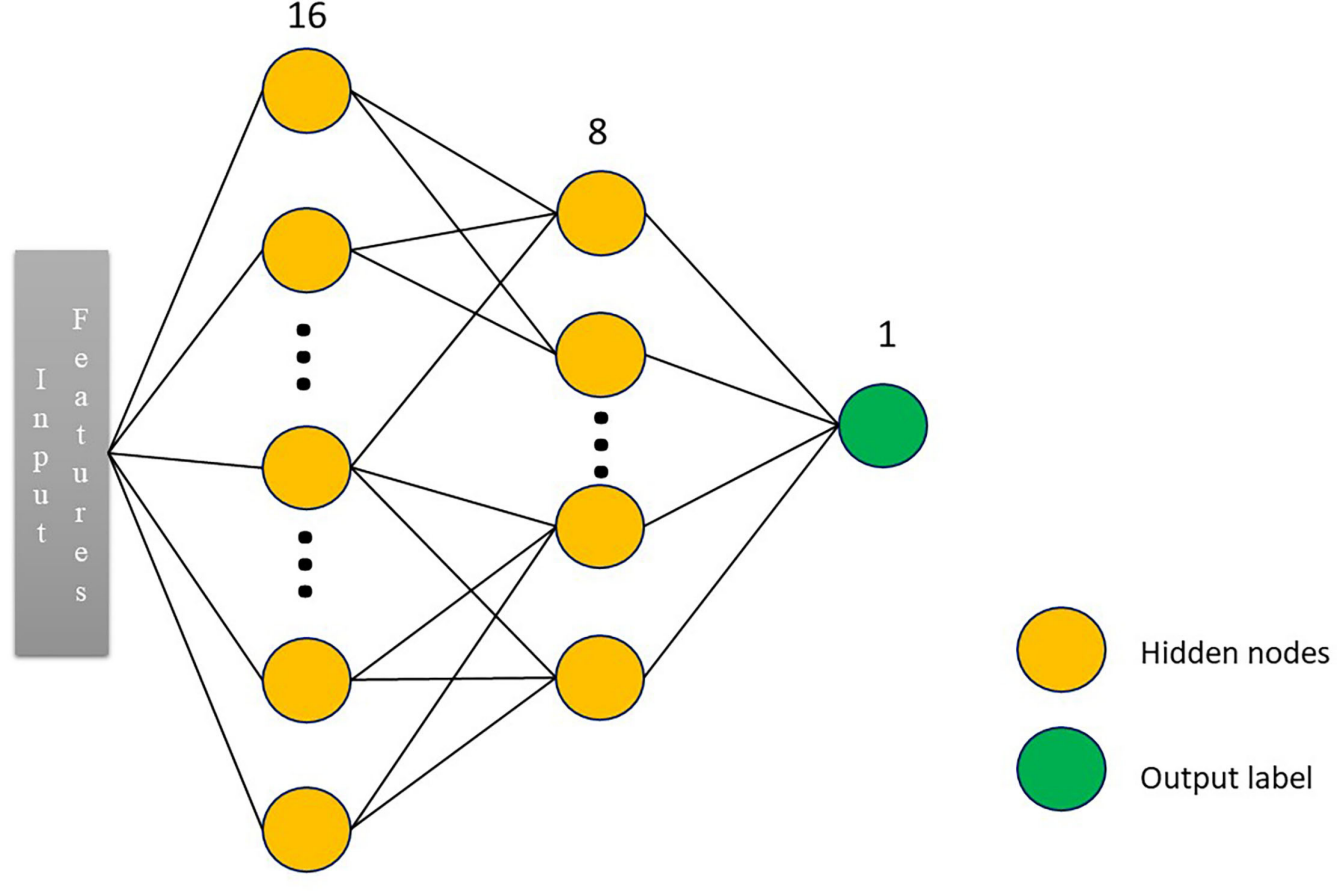
The overall architecture of the neural network. We constructed a three-layer multilayer perceptron (MLP) network classifier. The input was the radiomic features. The number of nodes for two hidden layers was set to 16 and 8, respectively. The number of nodes for output layer was set to 2, the same as the number of patient's group.

In this study, a four-layer convolutional neural network model, namely, multilayer perceptron (MLP) model, was constructed with the selected seven radiomic features, such as original_shape_Sphericity, original_firstorder_Median, original_firstorder_RobustMeanAbsoluteDeviation, original_glcm_ClusterShade, original_glcm_Imc1, original_gldm_DependenceNonUniformityNormalized, and original_glrlm_RunEntropy as input. MLP is an artificial neural network that has also performed well in the previous studies (20, 21). The overall architecture of MLP is shown in Figure 3. Random search was used to select the best configuration of hidden layer number nodes. The number of nodes for two hidden layers was set to 16 and 8, respectively. The number of nodes for output layer was set to 2, the same as the number of patient's group. The learning rate was 0.0001. The optimizer was stochastic gradient descent, and the loss function was the cross-entropy. Approximately 5-fold cross-validation was used in the training process. We derived this radiomics-based deep learning model using Pytorch 1.7.1 (https://pytorch.org), and we stopped training when the model converged. The model was trained with 150 epochs.

### End-to-end CNN Model and Visualization

We trained an 2D end-to-end CNN model that integrated an automatic feature extraction and a discriminative classifier into one model. The raw CT scans were the input and the classification label was the direct output. There were 1,138 glioma and 1,218 PCNSL slices in the training set. Transfer learning was used, and a pretrained Densenet-121 (22) model on ImageNet (http://www.image-net.org/) was fine-tuned to perform classification on our dataset. We converted our 2D slices into color images and resized them to the Densenet input size (224 × 224). Slices for each patient were classified by our trained model, and the final decision was performed by majority voting. The loss function was cross-entropy, and the optimizer was Adam. The learning rate was set to 0.0001 without weight decay. The batch size was 8. We applied 5-fold cross-validation and trained 500 epochs.

For the growing importance of interpretability in deep learning, we applied Grad-Cam++ (23) technique to draw coarse localization heatmaps, which highlighted the important regions for CNN model to make diagnosis decisions. The model paid more attention to the deeper red region.

### Physician's Diagnosis

In addition, all images were read by four independent radiologists, and three were primary radiologists (with 3, 4, and 4 years of experience in neuroradiology). One was a senior neuroradiologist (17 years of experience). The four independent radiologists were blinded to the initial diagnosis report, the pathological and clinical information, but were aware of that the segmented tumors were either PCNSL or glioma. The consistency of diagnosis from three readers was evaluated using intra- and interclass correlation coefficients (ICCs). An ICC of < 0.4 indicated poor correlation; an ICC of 0.4 to 0.75 indicated fair to good correlation; and an ICC of more than 0.75 indicated excellent correlation (24).

### Statistical Analysis

Area under the receiver operating characteristic curve (AUC) and accuracy (ACC) were used to evaluate the performance of the models. All statistical analyses were performed using Python (version 3.5.6). Categorical variables were presented as absolute numbers and counts with percentages. Summary statistics are presented as means ± standard deviation (SD) for normally distributed continuous variables, or as median [interquartile range (IQR)] for non-normally distributed continuous variables. Mann–Whitney *U* test was performed to compare continuous variables, whereas chi-squared test was used for categorical variables between groups. Delong tests was performed to compare the AUC of different models. A two-tailed *p* < 0.05 indicated statistical significance. ICC was used to evaluate the consistency between different readers.

The training and validation of the MLP and CNN models were implemented using Pytorch 1.7.1 (https://pytorch.org) with Tesla V100 GPU support.

## Results

### Study Population

In this study, the PCNSL cohort comprised of 50 patients (27 men and 23 women) with a mean age of 61.1 ± 12.1 years whereas the glioma cohort comprised of 51 patients (28 men and 23 women) with a mean age of 56.4 ± 13.0 years. The detailed patient demographics of the dataset are listed in Table 1. There were no significant differences between the PCNSL and glioma sets in terms of gender (*p* = 0.913), history of malignancy (*p* = 0.624), and tumor location (*p* = 0.066).

**Table 1:** Demographic and clinical characteristics of all patients.

**Characteristic**	**PCNSL**	**glioma**	***P* value**
Gender			0.913
Male	27 (54.0%)	28 (54.9%)	
Female	23 (46.0%)	23 (45.1%)	
Age, mean ± SD (years)	61.1 ± 12.1	56.4 ± 13.0	0.017
Tumor Location			0.066
Telencephalon	40	49	
Thalamus	5	2	
Brainstem	2	0	
Cerebellum	3	0	
History of malignancy			0.624
No	49 (98.0%)	48 (94.2%)	
Yes	1 (2.0%)	3 (5.8%)	

### Performance of Different Models

**Figure 4:**
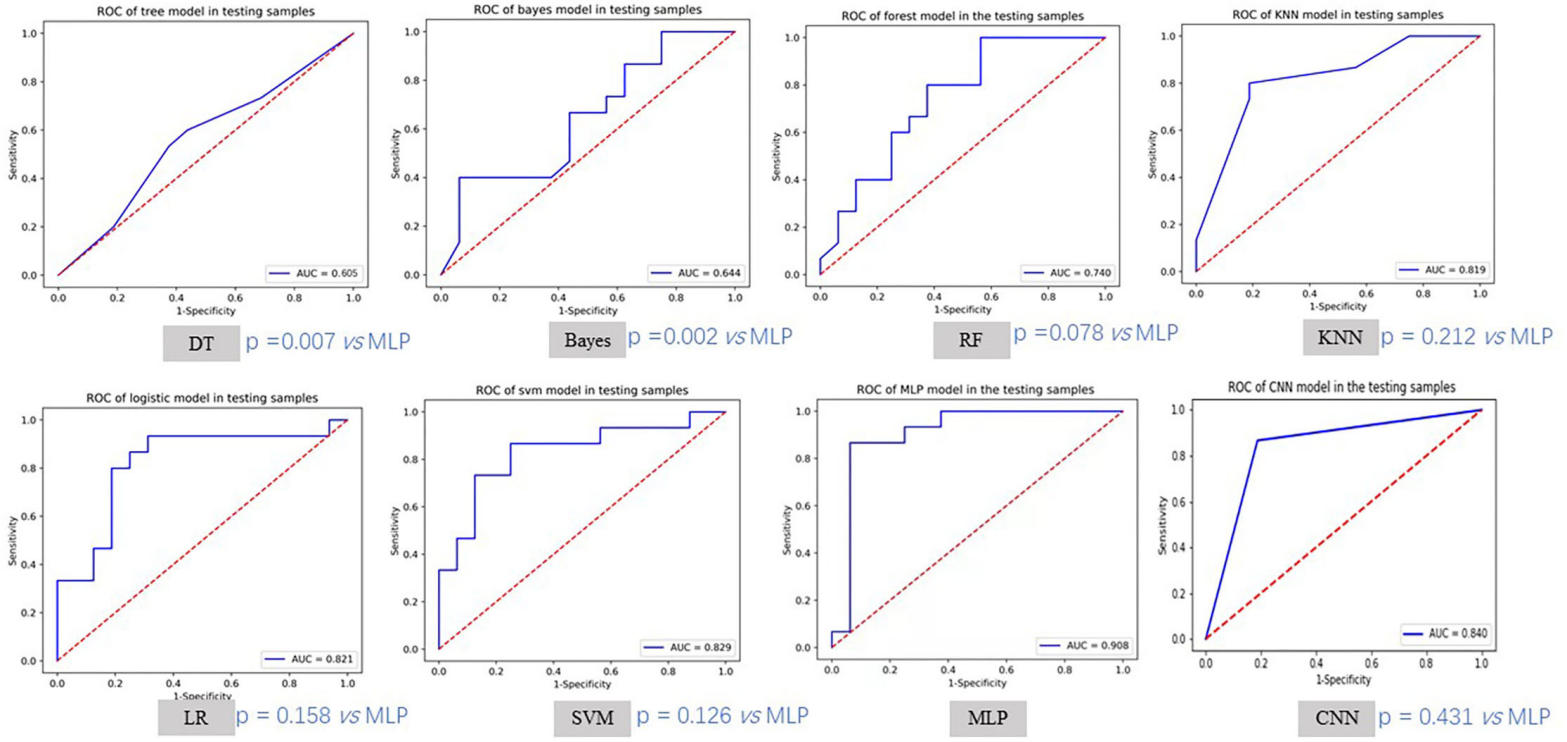
The ROC curves of different models in testing samples. MLP method achieved the best testing AUC. We also compared other methods with MLP using Delong test.

The training and validation performances of six machine learning models, MLP model, end-to-end CNN model, and four radiologists are presented in Table 2. Figure 4 demonstrated the area under the receiver operating characteristic curves (AUCs) of all models. Among all the machine learning models, RF had the best performance (AUC = 0.998, ACC = 0.957), followed by SVM (AUC = 0.930, ACC = 0.829), DT (AUC = 0.923, ACC = 0.900), LR (AUC = 0.885, ACC = 0.814), KNN (AUC = 0.852, ACC = 0.771), and naive Bayes (AUC = 0.796, ACC = 0.714) in the training set. In the validation set, the performance of SVM (AUC = 0.829, ACC = 0.742) was the best among the six RMs, followed by LR (AUC = 0.821, ACC = 0.774), KNN (AUC = 0.819, ACC = 0.774), RF (AUC = 0.740, ACC = 0.710), Bayes (AUC = 0.644, ACC = 0.581), and DT (AUC = 0.605, ACC = 0.608). There was obvious overfitting in DT and RF models. In comparing diagnostic performances, the ACCs of the three primary radiologists were 0.742, 0.710, and 0.710 for readers 1, 2 and 3, respectively. The ICC of the three radiologists was 0.651 (*p* < 0.001). The senior radiologist achieved an accuracy of 0.839, which was higher than the three primary radiologists.

**Table 2:** Performance of different models in training set and validation set.

**Method**	**Training**						**Validation**					
	**AUC (95%CI)**	**ACC (p)**	**Sensitivity**	**Specificity**	**PPV**	**NPV**	**AUC (95%CI)**	**ACC (p)**	**Sensitivity**	**Specificity**	**PPV**	**NPV**
LR	0.885	0.814 (0.810)	0.800	0.829	0.824	0.806	0.821	0.774 (0.470)	0.800	0.750	0.750	0.800
	(0.816–0.942)						(0.711–0.939)					
RF	0.998	0.957 (0.810)	0.943	0.971	0.971	0.944	0.740	0.710 (0.149)	0.800	0.625	0.667	0.769
	(0.995,1.0)						(0.577,0.880)					
SVM	0.930 (0.877–0.974)	0.829 (0.231)	0.857	0.800	0.811	0.848	0.829 (0.709–0.946)	0.742 (0.071)	0.867	0.625	0.684	0.833
DT	0.923	0.900 (0.151)	0.971	0.829	0.850	0.967	0.605	0.581 (0.470)	0.600	0.562	0.562	0.600
	(0.874–0.967)						(0.492–0.727)					
Naive Bayes	0.796	0.714 (0.632)	0.771	0.657	0.692	0.742	0.644	0.516 (0.988)	0.467	0.562	0.500	0.529
	(0.705–0.870)						(0.475–0.803)					
KNN	0.852	0.771 (0.031)	0.629	0.914	0.880	0.711	0.819	0.774 (0.988)	0.733	0.812	0.786	0.765
	(0.777–0.914)						(0.699–0.932)					
Radiologist1	-	0.743 (0.810)	0.714	0.771	0.758	0.730	-	0.742 (0.718)	0.733	0.750	0.733	0.750
Radiologist2	–	0.714 (0.810)	0.686	0.743	0.727	0.703	-	0.710 (0.470)	0.733	0.688	0.688	0.733
Radiologist3	–	0.729 (0.339)	0.771	0.686	0.711	0.750	–	0.710 (0.470)	0.600	0.813	0.750	0.684
Radiologist4	–	0.843 (1.000)	0.829	0.857	0.853	0.833		0.839 (1.000)	0.800	0.875	0.857	0.824
MLP	0.957	0.886 (0.473)	0.914	0.857	0.865	0.910	0.908	0.903 (0.988)	0.867	0.937	0.928	0.882
	(0.923,0.980)						(0.885,0.941)					
CNN	0.957	0.957 (0.632)	0.971	0.943	0.944	0.971	0.840	0.839 (0.470)	0.867	0.813	0.813	0.867
	(0.928–0.979)						(0.797–0.900)					

*AUC, area under curve; ACC, accuracy; PPV, positive predictive value; NPV, negative predictive value.*

*Radiologist4 is a senior neuroradiologist with 17 years' experience while the other three are senior neuroradiologists with less than 5 years' experience*.

*P is significant difference in accuracy compared to the senior radiologist4 using chi-squared test*.

The MLP performed best than other models, with an accuracy of 0.886 and 0.903, sensitivity of 0.914 and 0.867, specificity of 0.857 and 0.937, and AUC of 0.957 and 0.908 in the training and validation cohorts, respectively. There was no significant difference compared to the senior radiologist in accuracy (0.903 vs. 0.839, *p* = 0.988 using chi-squared test). The end-to-end CNN model achieved an accuracy of 0.957 and 0.839, sensitivity of 0.971 and 0.867, specificity of 0.943 and 0.813, and AUC of 0.957 and 0.840 in the training and validation cohorts, respectively, which had no significant difference in validation accuracy compared to the MLP model (*p* = 0.472) and the senior radiologist (*p* = 0.470).

**Figure 5:**
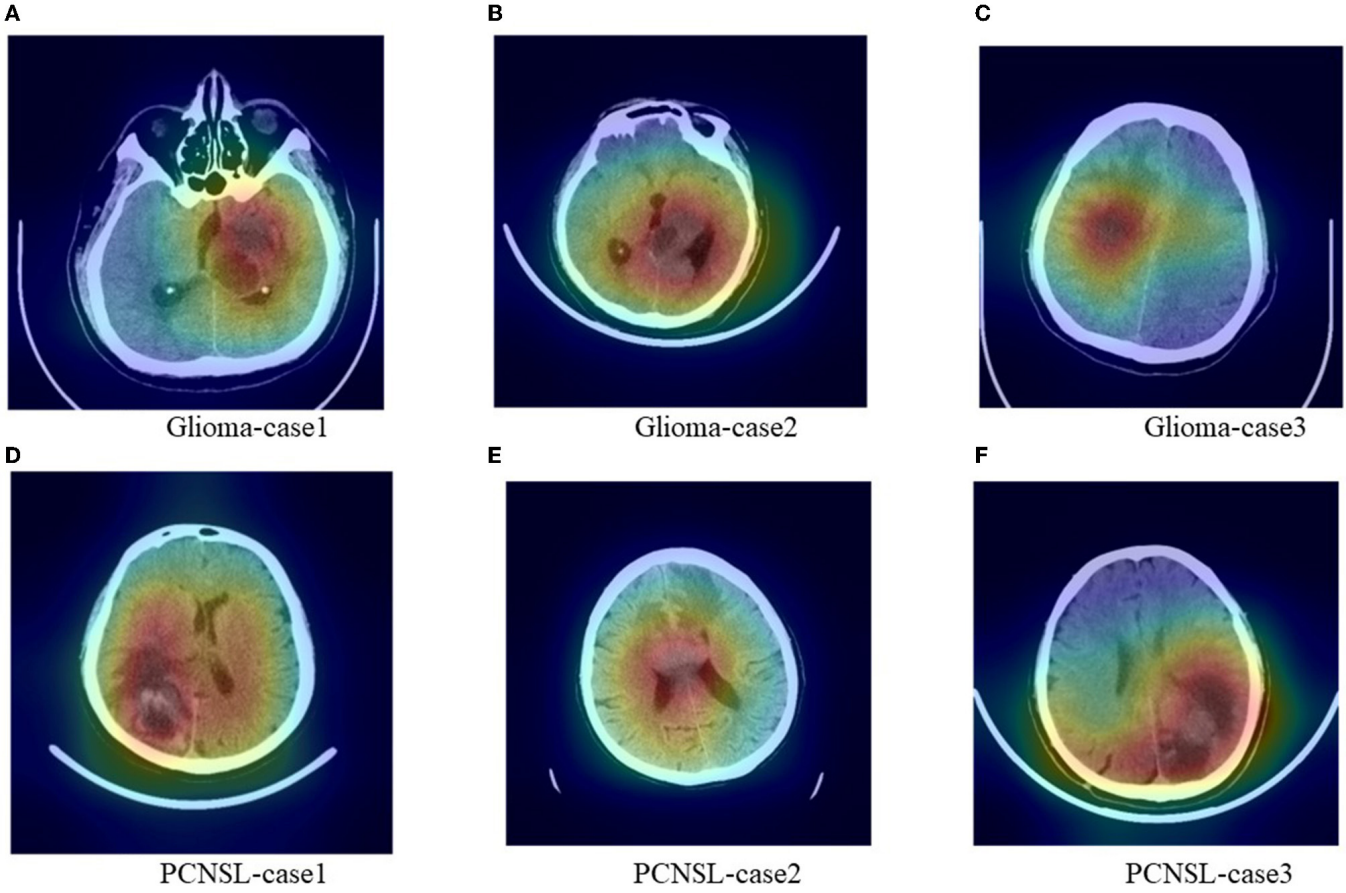
Representative images with heatmaps from the CNN model using Grad-Cam++ methods. The red regions were important for the diagnosis decision and the deep red regions overlapped with the tumor area. **(A–C)** were cases for glioma and **(D–F)** were cases for PCNSL.

We added Figure 5 which includes six cases Grad-Cam++ heatmaps that were obtained from the last convolutional layer in the network. The heatmaps might provide the most important regions that influenced the diagnosis decision. The deep red regions overlapped with the tumor area.

## Discussion

Differentiation between PCNSL and glioma is an important but challenging task. The question of “PCNSL” or “glioma” may become “resection or nonsurgical treatment” bypassing the steps of biopsy, histological evaluation, and postsurgical patient recovery. This would undoubtedly help expert neurosurgeons to optimize both patient outcomes and the cost-effectiveness of neurosurgical care (25). In this review, we explored various machine learning and deep learning methods based on radiomic features extracted from CT scans to predict PCNSL and glioma types and compare the performance of different models on 101 patients from five Chinese medical centers. Among all the models, the MLP model performed best, with an accuracy of 0.886 and 0.903, sensitivity of 0.914 and 0.867, specificity of 0.857 and 0.937, and AUC of 0.957 and 0.908 in the training and validation cohorts, respectively, and was significantly higher than the three primary physician's diagnoses (ACCs ranged from 0.710 to 0.742, *p* < 0.001 for all) and comparable with the senior radiologist (ACC 0.839, *p* = 0.988). The end-to-end CNN model achieved an AUC of 0.839 and an ACC of 0.840 in the validation cohort, which had no significant difference in accuracy compared to the MLP model (*p* = 0.472) and the senior radiologist (*p* = 0.470). The CNN model used raw CT scans as input. Feature extraction and selection were automated and implemented. The coarse localization heatmaps help to give an intuitive understanding of the mechanisms of the model.

Patients with PCNSL and glioma shared many similar clinical symptoms and overlapping imaging characteristics. In our study, the three primary physician's diagnoses results ranged from 0.710 to 0.742 and the experienced radiologist achieved 0.839. The fine-grained features of tumor regions were difficult to identify with the naked eyes. The low accuracy indicated the difficulties in identifying the two brain tumors with the gross visual. The studies reported that even using high-resolution MR sequences, visual differentiation of the two tumors was still challenging (26, 27). Radiomics enabled the conversion of original images into high-dimensional feature spaces that allowed an improved performance in PCNSL and glioma. Radiomic ML algorithms performed as well as or better than radiologists in several studies (14, 28, 29). Bathla et al. (17) compared the predictive performance of various ML techniques to differentiate between PCNSL and glioma using a combination of various feature selection and ML algorithms on 94 patients, and several models achieved comparable performance.

The MLP model is a type of feed-forward artificial neural network model that has the input and output layers connected by a hidden layer. It helps to evaluate nonlinear relationships in classification tasks (30) and improve efficiency (31). Yun et al. (16) established an MRI radiomic MLP model to distinguish PCNSL from glioma and found high performance, which was even better than unsupervised convolutional neural networks. In our study, among all of our machine learning methods, the nonlinear classifier SVM ranked first, which indicated that a more complex model was needed for the input features. Therefore, we established an MLP classifier that had the capability of producing a higher level and more abstracted feature selection algorithm and it performed the best.

Convolutional neural network model is an automatic model for differentiating between PCNSL and glioma. It was built on raw CT slices and required no segmentation of tumor region. Training from scratch was usually data hungry, so transfer learning was applied. Natural images shared some underlying features with medical images and the pretrained model helped to improve the convergence rate. The validation results showed that CNN model had no significant difference in accuracy compared to the senior radiologists. According to the heatmaps, the red important regions overlapped with the tumor regions, which showed that the diagnosis decision of CNN mode was based on the tumor rather than background regions. The effectiveness and reasonability have been proved.

Although the MRI is the recommended check for brain tumor diseases diagnose and it has a better resolution in brain tissue, cost-effective CT is routinely performed for patients in clinical practice. In our cases, there are final pathologically confirmed patients with PCNSL unable to accept prepuncture due to the tumor location and mistakenly underwent surgery. CT has its own advantages, such as short scan time and low price. Also, CT is performed for some patients who cannot have MR check, such as people with implantable medical devices, including pacemakers and cochlear implants. The ML or DL algorithms have achieved great success in differentiating between PCNSL and glioma on MR scans in previous studies and inspired us to verify distinguish on CT scans. To our knowledge, the effectiveness of CT-based ML or DL models for predicting the two tumors has been rarely explored before. Therefore, we collected data from multicenter and established different models to explore the performance on the two tumors classification tasks. We followed the standard radiomic process: ROI segmentation, feature selection, and model establishment and validation. An effective feature selection is a crucial step because radiomic features are multiple collinear and correlated predictors that could produce unstable estimates and might overfit predictions. In our models, 7 features remained after feature selection, and using fewer features was a good strategy against overfitting. In this study, MLP model was significantly higher than the three primary physician's diagnoses and the six machine learning methods. CNN model made comparable prediction result with MLP method (*p* = 0.431 in AUC) and senior radiologist (*p* = 0.470 in ACC). CNN was also recommended since the whole process rarely required human intervention. The multicenter study validated the robustness and generalizability of our model, and it could provide a convenient and accurate tool for radiologists to identify PCNSL and glioma types on CT scans.

Besides the retrospective nature, our study also had several limitations. First, we used a relatively small number of patients. The small sample size could limit the effectiveness of supervised machine learning and deep learning methods and caused overfitting. It was quite possible that the model performance might vary with additional training data. We collected data from five Chinese medical centers and divided all data into training and validation sets by a ratio of 7:3. Unlike glioma, there was no free available PCNSL dataset which could be readily used. The absence of an external validation set could not help us to determine the generalizability of our findings. Nevertheless, our methods for CT radiomic feature analysis showed the potential to be reproducible with other datasets, although our classifiers might exhibit limited value. Second, the proposed models were used especially for the discrimination of PCNSL and glioma, whereas in clinical practice, the single brain metastasis could show similar appearance on CT which might cause diagnostic difficulties. Further study with collecting more data to validate the generalizability of the developed models and a more advanced multiclassification model needs to be explored. Our classifiers could serve as a base model for the discrimination of PCNSL and glioma and might have the potential to be an effective aided diagnostic tool for clinical practice to some extent. If more kinds of brain tumor data could be collected, a baseline model could be conducted quickly.

In conclusion, we established machine learning and deep learning models from CT scans to help to differentiate PCNSL and glioma and verified that the models were feasible and provided high-performance -like senior physician's diagnoses, which showed the potential to assist clinical decision-making.

## Data Availability Statement

The datasets presented in this article are not readily available because of privacy and ethical concerns. Requests to access the datasets should be directed to mvvmouweiwei@126.com.

## Ethics Statement

Written informed consent was not obtained from the individual(s) for the publication of any potentially identifiable images or data included in this article.

## Author Contributions

Guarantor of integrity of the entire study and literature research: WM. Definition of intellectual content: LM. Experimental studies: GL and WM. Data acquisition: GL and YZ. Data analysis and statistical analysis: WW and GL. Paper writing: All authors. All authors contributed to the article and approved the submitted version.

## Conflict of Interest

Author WW was employed by GE Healthcare. The remaining authors declare that the research was conducted in the absence of any commercial or financial relationships that could be construed as a potential conflict of interest.

## Publisher's Note

All claims expressed in this article are solely those of the authors and do not necessarily represent those of their affiliated organizations, or those of the publisher, the editors and the reviewers. Any product that may be evaluated in this article, or claim that may be made by its manufacturer, is not guaranteed or endorsed by the publisher.
